# Amikacin Dosing Adjustment in Critically Ill Oncologic Patients: A Study with Real-World Patients, PBPK Analysis, and Digital Twins

**DOI:** 10.3390/pharmaceutics17030297

**Published:** 2025-02-24

**Authors:** Juliana Queiroz da Silva, Natália Valadares de Moraes, Rita Estrela, Diogenes Coelho, Diego Feriani, Karen Migotto, Pedro Caruso, Ivan Leonardo França e Silva, Daiane de Araujo Oliveira, João Paulo Telles, Fernanda de Lima Moreira

**Affiliations:** 1 Laboratory of Pharmacometrics (LabFarma), Faculty of Pharmacy, Federal University of Rio de Janeiro, Rio de Janeiro 21941-902, Brazil; 2 Center for Pharmacometrics and Systems Pharmacology, Department of Pharmaceutics, College of Pharmacy, University of Florida, Orlando, FL 32827, USA; 3 Evandro Chagas National Institute of Infectious Diseases INI, Fiocruz, Rio de Janeiro 21040-360, Brazil; 4 Department of Infectious Diseases, AC Camargo Cancer Center, São Paulo 01509-010, Brazil; 5 Department of Pharmacy, AC Camargo Cancer Center, São Paulo 01509-010, Brazil; 6 Department of Intensive Care Medicine, AC Camargo Cancer Center, São Paulo 01509-010, Brazil

**Keywords:** MIPD, dose adjustment, pharmacokinetics, aminoglycoside, nephrotoxicity

## Abstract

**Background/Objectives:** Guidelines recommend adjusting amikacin dosing based on patients’ renal function. Nevertheless, for critically ill cancer patients, the renal function equations based on serum creatinine levels have low or no correlation with amikacin clearance. Considering this, using real-world data, we built an amikacin PBPK model to predict amikacin plasma concentrations in critically ill oncologic patients stratified by renal impairment levels. Further, the model was applied for dose stratification and individualization (digital twin strategy) in this population. **Methods:** In the Therapeutic Drug Monitoring (TDM) study, 368 amikacin pharmacokinetic analyses from 184 critically ill cancer patients were enrolled in three cohorts. A full-body PBPK model was developed using PK-Sim v. 11.3. **Results:** The final PBPK model accounted for two groups of critically ill cancer patients with mild (creatinine clearance; CLcr ≥ 60 mL/min) or severe (CLcr < 60 mL/min) renal dysfunction. In the dose stratification strategy, at the 7th dose, cancer patients with CLcr ≥ 60 mL/min under regimens 20 mg/kg (q24h); 25 mg/kg (q24h); 25 mg/kg (q48h); and 30 mg/kg (q72h) have probability of ≥69% of the patients achieving the efficacy target (AUC/MIC > 80, MIC of 4 mg/L), while cancer patients with CLcr < 60 mL/min under regimens 7.5 mg/kg (q24h); 15 mg/kg (q24h); 15 mg/kg (q48h); and 20 mg/kg (q36h) have ≥90% probability of achieving the same efficacy target. **Conclusions:** Our MIPD approach demonstrates potential in optimizing amikacin dosing for critically ill cancer patients. However, it does not eliminate the need for TDM due to unexplained variability still not accounted for by the PBPK model.

## 1. Introduction

Amikacin, an antimicrobial aminoglycoside, is widely used in hospitals to treat severe bacterial infections due to its broad-spectrum activity against Gram-negative pathogens and resistance to aminoglycoside-inactivating enzymes [1]. However, its use is associated with a high risk of nephrotoxicity and therapeutic failure, making the optimization of amikacin dosage regimens crucial [2]. Amikacin-related nephrotoxicity is dose-dependent [3], and a target minimum plasma concentration (C_min_) lower than 2.5 to 5 mg/L has been proposed to mitigate this risk [4,5].

Amikacin is a hydrophilic drug with low protein binding and distributes primarily to extracellular fluids. In healthy volunteers, the mean volume of distribution (Vd) for amikacin is 11 L [6], while in critically ill patients, it is higher, with a mean Vd of 18.6 L, ranging from 10.7 to 41.5 L [7]. Amikacin is primarily eliminated unchanged through glomerular filtration in the kidneys. In healthy volunteers, the mean clearance (CL) for amikacin is 4 L/h [6], whereas critically ill patients have a higher mean clearance of 5.2 L/h, with a range of 4.4 to 12.1 L/h [7]. For critically ill cancer patients, amikacin clearance ranges from 1.08 to 3.36 L/h [8].

International guidelines recommend adjusting amikacin dosing based on patients’ renal function [9]. However, the dosage recommendations vary among the guidelines [10,11,12,13]. Our previous study applied different renal function equations based on serum creatinine levels to evaluate the correlation with amikacin clearance in critically ill cancer patients [8]. None of the eight renal function formulas tested, including the Cockroft–Gault formula, had an appropriate correlation with the amikacin clearance in this population, probably because the cancer-related pathophysiological changes were not included in the building processes of these formulas. Consequently, current dosage guidelines may not apply to this special population, highlighting the need for new strategies to achieve optimal amikacin therapy in oncologic patients.

Model-informed precision dosing (MIPD) aims to improve pharmacotherapy outcomes through individualized treatment based on the patient’s characteristics [14]. In this context, physiologically based pharmacokinetic modeling and simulation (PBPK M&S) is a valuable tool for optimizing dose regimens in stratified populations, incorporating specific population characteristics. Recently, the digital twins approach using PBPK M&S has shown promise for dose individualization, using patient individual characteristics as input data, such as demographics, genetics, liver and kidney functions [15,16].

In our previous study [8], we developed an amikacin PBPK model stratifying the population by renal function status. Amikacin clearance in oncologic patients did not match the expected clearance according to the renal impairment classification [17]. Using real-world data, we updated the previous amikacin PBPK model using the open-source PK-Sim^®^ platform to predict amikacin plasma concentrations in critically ill oncologic patients stratified by renal impairment levels. Further, this updated PBPK model was applied for this population’s dose stratification and individualization (digital twin strategy).

## 2. Methods

### 2.1. Clinical Protocol, Ethical Aspects, and Sample Collection

The protocols for conducting amikacin therapeutic drug monitoring (TDM) study were previously described by Telles et al. [8]. The clinical protocol was approved by the Research Ethics Committee of AC Camargo Cancer Center (CAE 56419722.0.0000.5432). This retrospective study evaluated three different cohorts of critically ill cancer patients, summing 189 patients evaluated. In cohort #1, 92 blood samples from 51 patients were collected for internal validation of the PBPK model. In cohort #2, 98 patients were assessed with 179 blood samples collected for external validation of the PBPK model. For the digital twin strategy, cohort #3 with 40 patients was evaluated with 80 blood samples collected.

### 2.2. Physiologically Based Pharmacokinetic Model Development

**Figure 1:**
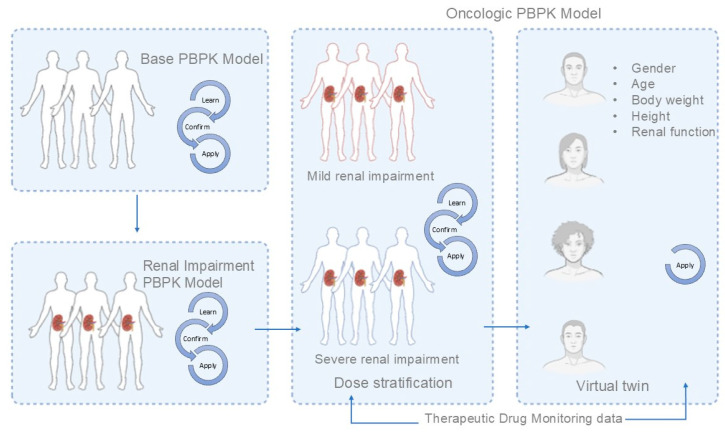
Amikacin PBPK model development, validation, and application workflow.

A full-body PBPK model for amikacin was developed using PK-Sim^®^ software version 11.3 (Bayer Technology Services, Leverkusen, Germany). A schematic workflow of the model development, validation, and application is depicted in Figure 1. The physicochemical and absorption, distribution, metabolism, and excretion (ADME) input data for amikacin are detailed in Table S1. The strategy for developing the amikacin PBPK model involved transposing our previous model [8] built in Simcyp v. 21 (Certara, Princeton, NJ, USA) to the open-source PK-Sim platform. The input data was the same as previously reported [8], and the distribution model also utilized the Rodgers and Rowland equation.

However, similar to our previous model, the specific organ permeability calculated by the Rodgers and Rowland model did not match the observed plasma concentrations in cancer patients. Therefore, this value was adjusted using the parameter identification tool to accurately describe amikacin disposition in the cancer population. Different from our previous model [8], amikacin renal clearance was estimated by the glomerular filtration rate (GFR) method in PK-Sim, with the GFR fraction to 1.0, indicating glomerular filtration process in amikacin elimination.

### 2.3. Amikacin PBPK Model Validation

Amikacin plasma concentration versus time profiles were extracted using the web-based data extraction tool WebPlot Digitizer (https://automeris.io/WebPlotDigitizer/) (accessed on 12 August 2024) and used for model validation. Initially, the PBPK model was verified and validated based on the design of clinical trials previously published for healthy volunteers, followed by evaluation in critically ill non-cancer patients with varying stages of renal dysfunction (Table S2) [6,18,19,20,21]. The PBPK model verification was performed using visual predictive checks, and the ratios of observed to predicted pharmacokinetic parameters AUC, C_max_, CL, and Vd within a 2-fold error range (0.5 ≤ ratio ≤ 2) were considered adequate.

### 2.4. Renal Impairment Population Model

During the modeling process, populations were initially stratified based on the estimated creatinine clearance (eGFR) based on Cockroft–Gault formula, following FDA guidelines [17] for renal function classification: normal renal function (eGFR > 90 mL/min/1.73 m^2^); mild renal impairment (eGFR 60–89 mL/min/1.73 m^2^); moderate renal impairment (eGFR 30 to 59 mL/min/1.73 m^2^); and severe renal impairment (eGFR 15 to 29 mL/min/1.73 m^2^). Key physiological parameters, including plasma protein, hematocrit, renal blood flow, hepatic blood flow, kidney volume, and albumin, were considered for building these populations (Table S3), based on Wu et al. [22] and Heimbach et al. [23].

### 2.5. Oncologic Population with Renal Impairment Model

**Figure 2:**
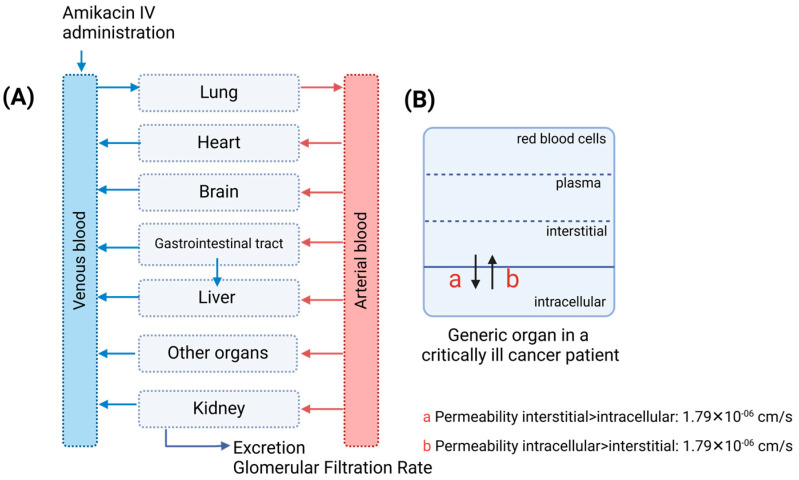
Schematic of the amikacin full PBPK model in critically ill oncologic patients. (**A**) Full physiologically based pharmacokinetic model; (**B**) Compartments included in each organ or tissue with the increased intracellular–interstitial permeability observed in critically ill oncologic patients.

Figure 2 shows a scheme of the model components of the amikacin PBPK model in an oncologic population with renal impairment. To fit the observed data, the specific organ permeability value was optimized to 1.79 × 10^−6^ cm/min (from an initial value of 1.79 × 10^−15^ cm/min). This change in the model represents an increased amikacin distribution to tissues in critically ill patients with renal impairment compared to healthy volunteers [8,21].

To further refine the amikacin PBPK model in cancer patients, observed plasma concentrations were normalized by dose. Critically ill cancer patients were initially stratified according to FDA [17] renal function classifications: normal function, mild, moderate, and severe renal dysfunction. A sensitivity analysis was conducted to evaluate which input parameters impacted amikacin plasma clearance in cancer patients.

The sensitivity was calculated as follows:

$$S=\frac{∆PK}{∆p}\;\times \;\frac{p}{PK}$$

where *S* is the sensitivity, *PK* is the initial value of the pharmacokinetic parameter, Δ*PK* is the change of the pharmacokinetic parameter from the initial value, *p* is the initial value of the examined parameter, and Δ*p* is the change of the examined parameter from the initial value, respectively. A sensitivity of +1.0 indicates that a +10% change of an examined input parameter causes a +10% change in the predicted pharmacokinetic parameter. A sensitivity analysis, including all input data, was generated, and only parameters higher than +0.5 were considered relevant. The data were expressed as mean and 95% lower and upper confidence interval (95%CI).

In the next step, a simpler model development strategy was adopted aiming for the ready application in clinical practice. For this, the creatinine clearance (CLcr) cutoff point value of 60 mL/min, dividing the critically ill cancer patients from cohorts #1 and #2 into two groups for simulation using the oncologic PBPK model: (1) Critically ill cancer patients with mild renal impairment, considering CLcr ≥ 60 mL/min; (2) Critically ill cancer patients with severe renal impairment, considering CLcr < 60 mL/min.

To adjust the amikacin elimination in those groups, the parameter identification tool provided by PK-Sim^®^, using the Leverenberg–Marquardt algorithm, was applied to find the optimal eGFR value within a specific range to minimize the residuals between the simulation output and the actual observed values from the TDM study. Amikacin’s observed plasma concentrations from the internal validation group (cohort #1) were used in this simulation to set the eGFR value for each group. Further, the observed amikacin plasma concentrations from the external validation group (cohort #2) were used to verify the model adjustment.

The eGFR values and physiological changes used for building the critically ill cancer population stratified into two groups are demonstrated in Table 1. The coefficient variation for the eGFR value was set at 70% for both groups to represent the interindividual variability observed in the TDM study accounting cohorts #1 and #2.

The digital population considered the mean demographic values of the proportion of females and range of age, weight, and height from cohorts #1 and #2 and simulated 100 patients. (1) Critically ill cancer patients with mild renal impairment—42% female, age of 20 to 81 years old, body weight of 41.6 to 132 kg; height of 144 to 194 cm; and a mean eGFR value of 63 mL/min/1.73 m^2^ (distribution of eGFR values in the population depicted in Figure S1, left panel); (2) Critically ill cancer patients with severe renal impairment—44% female, age of 27 to 89 years old, body weight of 44 to 100 kg; height of 145 to 190 cm; and a mean eGFR value of 18 mL/min/1.73 m^2^ (distribution of eGFR values in the population depicted in Figure S1, right panel).

#### Optimizing Amikacin Doses in Cancer Patients

The developed PBPK model was applied to optimize amikacin doses for oncologic patients according to the renal function groups previously described. Different scenarios with varying amikacin dosage regimens administered by intravenous infusions were simulated. Digital populations of cancer patients with mild or severe renal impairment, as previously described (Section 2.5), were simulated with 100 subjects each. Seven doses were simulated at 24, 36, 48, or 72 h dosing intervals. The C_max_, C_min_, and AUC at 7th dose were used to propose cancer patients’ doses stratified according to renal impairment. The response to amikacin was expressed as the percentage of target attainment, considering as a primary PK/PD index the area under the concentration curve (AUC) in the interval of drug administration divided by the minimum inhibitory concentration (MIC) above or equal to 80 (AUC/MIC ≥ 80) [4]. An alternative PK/PD index was the maximum plasma concentration (C_max_) divided by MIC ≥ 8 (C_max_/MIC ≥ 8) [4]. The MIC values considered for calculation were 4 mg/L and 8 mg/L. The percentage of patients with minimum drug concentration (C_min_) ≤ 5 mg/L was recorded, as this is considered to be safe regarding renal toxicity [4].

### 2.6. Application of the PBPK Model to Optimize Amikacin Doses Using a Digital Twin Approach

Forty individual digital twins were built in PK-Sim^®^ employing the matching data regarding sex, age, weight, and height of the patients. The renal dysfunction model was selected for each patient depending on the CLcr calculated by the Cockroft–Gault formula and considering the cutoff point of 60 mL/min. Each subject was simulated to match the amikacin dosing regimen of their respective twin. Plasma concentration versus time profile was compared with the individual patient’s data to verify the applicability of the developed model in individualizing the amikacin dosage regimen. The overall performance of the PBPK model was evaluated using a goodness-of-fit plot for the predicted versus observed plasma concentrations and magnitude of amikacin concentrations residual error versus time plot. The residuals were calculated using the following formula:

log(Simulation value) − log(Observed value) = log(Simulation Value/Observed Value)(1)

## 3. Results

Table 1 depicts the demographic and pathophysiological characteristics of the oncologic patients from cohorts #1 and #2 of the TDM study used for amikacin PBPK model development and validation.

The amikacin PBPK model was developed using PK-Sim^®^ software version 11.3 using the input parameters depicted in Table S1. The simulations generated with the “PBPK base model” adequately described the observed concentrations in healthy volunteers (Figure S2 and Table S4). Subsequently, the “Renal impairment PBPK model”, which accounts for varying degrees of renal dysfunction, was integrated into the population model. This approach demonstrated robust performance in accurately simulating plasma concentration–time profiles across critically ill patients with different levels of renal impairment (Figure S3 and Table S5). This suggests that the model is well-suited for capturing the altered pharmacokinetics in this population, potentially enhancing the dose stratification and/or individualization.

The patients from the internal validation data set (cohort #1, *n* = 46) (Table 2) were employed for building the “Oncologic PBPK model”, the external validation data set (cohort #2, *n* = 98) was employed for verification and refinement of the model. Initially, a sensitivity analysis evaluating the impact of all input data on amikacin systemic clearance in critically ill cancer patients (Figure S4) demonstrated that kidney volume, plasma protein scale factor, GFR fraction, and fraction unbound are the main parameters influencing this PK parameter. These input parameters and another related to AKI were considered for building the critically ill cancer population (Table 2).

**Figure 3:**
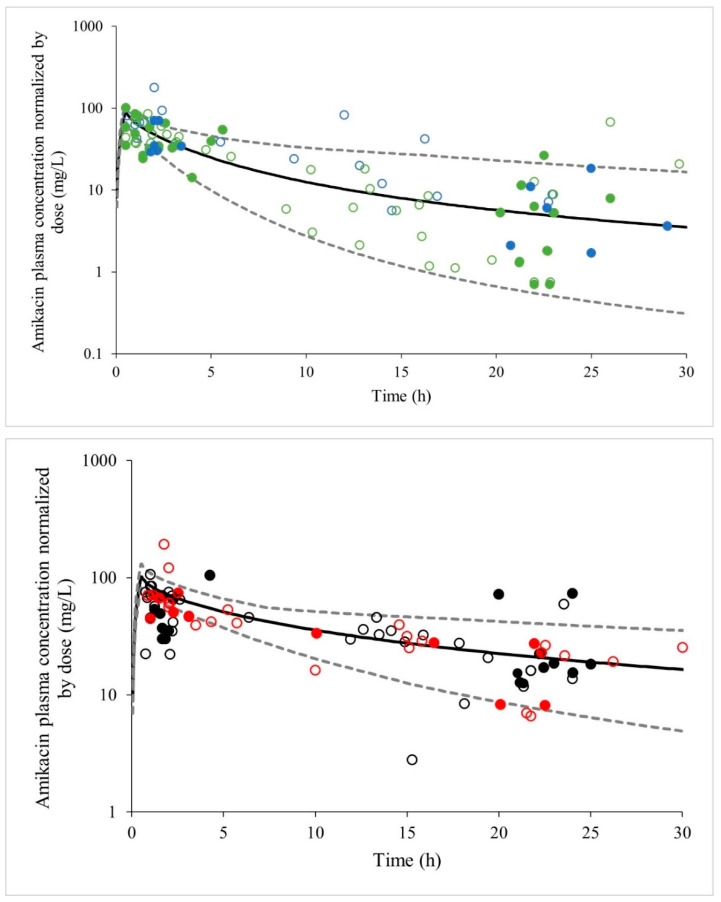
Plasma concentration versus time profiles of amikacin following a single IV infusion normalized dose of 1400 mg during 30 min for cancer patients with different stages of renal dysfunction. The solid line represents the simulated mean plasma concentration versus time model and the dashed lines represent the simulated 5th and 95th percentiles with the final PBPK. **Upper panel** represents critically ill cancer patients with mild renal dysfunction. The circles represent the observed data, and the patients were classified according to FDA guidance. Closed and open green circles represent internal and external validation data for normal renal function, respectively. Closed and open blue circles represent internal and external validation data for mild renal dysfunction, respectively. **Lower panel** represents critically ill cancer patients with severe renal dysfunction. Closed and open black circles represent internal and external validation data for moderate renal dysfunction, respectively. Closed and open red circles represent internal and external validation data for severe renal dysfunction, respectively.

In this scenario, the predicted plasma concentrations versus time profile of oncologic patients with mild renal dysfunction (Figure 3—upper panel) reasonably matched the amikacin exposure in oncologic patients from the TDM study with CLcr value ≥ 60 mL/min. Similarly, the predicted concentration–time profile for oncologic patients with severe renal dysfunction (Figure 3—lower panel) closely matched the amikacin exposure observed in the TDM study for oncologic patients with a CLcr value < 60 mL/min. However, greater variability was noted within these groups. For cancer patients with mild and severe renal impairment, the predicted Volume of Distribution (Vd) values, expressed as mean (confidence interval 5–95th), were 0.52 (0.42–0.61) L/kg and 0.42 (0.37–0.47) L/kg, respectively. The predicted clearance values for mild and severe renal impairment were 0.05 (0.01–0.10) L/h/kg and 0.01 (0.004–0.03) L/h/kg, respectively.

**Figure 4:**
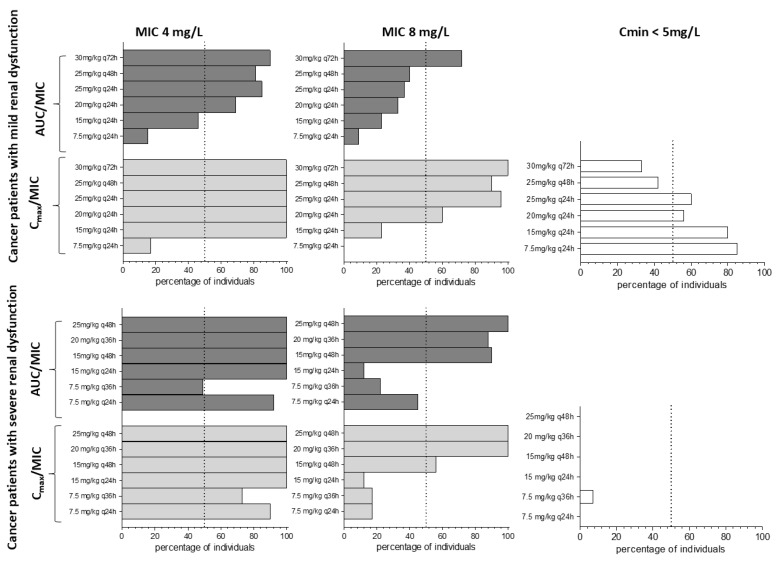
Amikacin posology efficacy and safety performance in the critically ill cancer population. Application of the final amikacin PBPK model to predict the percentage of cancer patients to attain the treatment target for efficacy (MIC 4 mg/L and MIC 8 mg/L) and safety (C_min_ ≤ 5 mg/L) at 7th dose. Critically ill cancer patients with mild renal dysfunction are demonstrated in the **upper panel** and critically ill cancer patients with severe renal dysfunction are shown in the **lower panel**. The filled bold dashed rectangles represent the PK-PD target attainment of C_max_ ≥ 8-fold MIC value; the filled short dashed rectangles represent the PK-PD target of AUC ≥ 80-fold MIC value. The white rectangles represent the safety target attainment considering C_min_ ≤ 5 mg/L. Legend: C_min_—concentration just before administering the 7th dose; MIC—minimal inhibitory concentration; AUC—Area under the curve in the dose interval at the 7th dose; C_max_—Maximum plasma concentration at the 7th dose.

Dosage regimens were proposed for cancer patients stratified according to renal function and evaluated as PK/PD targets for amikacin efficacy and safety (Figure 4). For mild and severe renal impairment in cancer patients, the dosage regimens simulated had higher efficacy rates when evaluating the C_max_ value than the AUC value as the PK parameter in the PK/PD index (Figure 4).

For mild renal impairment, the regimen composed of 15 mg/kg (q24h) had an ~80% rate of success in safety attainment, but less than 50% rate of success for efficacy attainment (AUC/MIC) considering MIC values of 4 and 8 mg/L. For severe renal impairment, the lower dosage regimen of 7.5 mg/kg (q36h) simulated produced a safety target attainment rate low (~10%), while the other dosage regimens simulated demonstrated that the severe renal dysfunction group is not protected from nephrotoxicity concentrations of amikacin, considering the C_min_ < 5 mg/L as the safety target.

**Figure 5:**
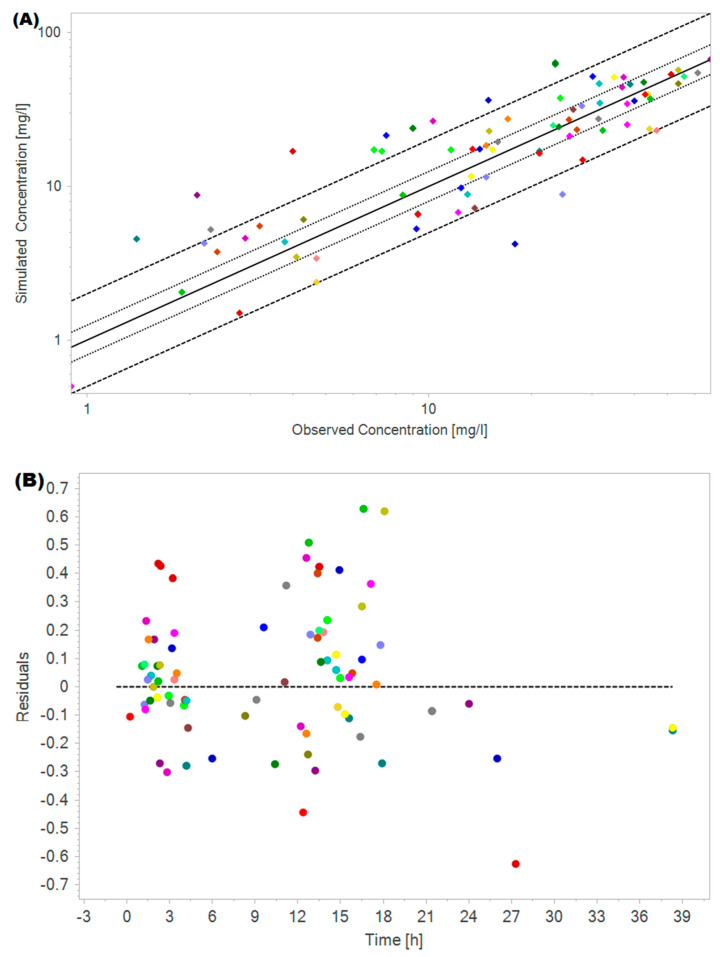
Performance evaluation of digital twin strategy employing the oncologic PBPK model for amikacin in predicting amikacin plasma concentrations from TDM study (*n* = 40). (**A**) The plot of the model generated versus observed concentration along with the identity line. The solid line indicates the line of unity, the dashed lines indicate a 2-fold range, and the dotted lines indicate a 1.25-fold range. The diamonds represent observed amikacin plasma concentration, and each color represents a different patient. (**B**) Amikacin concentrations residual error versus time plot. The circles represent observed amikacin plasma concentration, and each color represents a different patient.

Table 3 demonstrates the individual characteristics of critically ill cancer patients (*n* = 40, Cohort #3) used in the digital twin strategy employing the amikacin PBPK model. Figure 5 demonstrates the overall quality of the fit for the digital twin dataset with the amikacin oncologic PBPK model. It is observed that 83.8% of fold error values were within the two-fold range. Figure S5 shows the observed versus simulated amikacin plasma concentrations versus time for each matched pair of critically ill cancer patients from the TDM study cohort #3 (*n* = 40) and their respective digital twins obtained with the final PBPK model developed for critically ill cancer patients with renal dysfunction.

## 4. Discussion

In this study, we explored the impact of renal dysfunction degrees in critically ill cancer patients on amikacin exposure. Using the amikacin PBPK model developed for this population, we proposed dosage regimens based on typical amikacin clearances in the stratified population according to mild or severe renal impairment degree stated from serum creatinine data and the Cockroft–Gault formula. In the last step, we evaluated the power of our model to personalize the amikacin dosage regimen using a digital twin strategy.

During the PBPK model extrapolation from healthy volunteers to critically ill cancer patients, a considered increase in organ-specific permeability settled the increased Vd observed in the cancer population. Physiologically, this process could be explained by the endothelial dysfunction observed in critically ill patients that can correlate with higher capillary leakage [24] of the hydrophilic drug amikacin, explaining the higher Vd value observed in this population compared to healthy volunteers [7]. The organ-specific permeability value set in the present model is a non-mechanistic approach. An increase in plasma leakage to the interstitial space and input of a vasodilation process could be used for a mechanistic distribution model approach. Our final PBPK model differs from other amikacin PBPK models published in the literature [8,25,26] in its use of personalized input data for critically ill cancer patients model building. Specifically, we stratified critically ill cancer patients based on their degree of renal dysfunction, classifying them as having mild or severe renal impairment, as detailed in Table 1.

The increasing incidence of acute kidney injury (AKI) in the context of cancer has been reported [27,28], with an incidence rate of 24% to 54% [29,30] and a high mortality risk in critically ill cancer patients [31]. AKI may occur because of the cancer itself, and its treatment is associated with severe complications such as sepsis [32]. In this study, we evaluated critically ill cancer patients (Table 1), from a TDM study, with a mean creatinine clearance of 50.6 to 57.7 mL/min, indicating an overall decreased renal function. Furthermore, patients were undergoing treatment with nephrotoxicity drugs besides the well-known nephrotoxic amikacin. Among the co-administered nephrotoxic drugs, the incidence in the three TDM cohorts of furosemide, vancomycin, and polymixin use were 72.5–87%, 57.5–76%, and 7.5–21.7%, respectively. Comorbidities associated with renal impairment were at high incidence in the three TDM cohorts, with diabetes and hypertension affecting 21–32.5% and 12.5–47.8% of the patients, respectively. These concomitant diseases and medication alongside each patient’s cancer inflammation status may contribute to decreased renal function.

Some patient characteristics from the present TDM study can be compared with two other published studies [33,34]. The first one, ref. [33], evaluated the terminal stage of hematological cancer patients stratified as cachexia and non-cachexia. The second one, Aquino et al. [31], evaluated cancer patients stratified as neutropenic and non-neutropenic. The CLcr in those studies [33,34], measured using the Cockroft–Gault equation, varied from 50.6 to 89.3 mL/min, indicating that the overall cancer population under amikacin treatment has diminished renal function. The average dose administered in the present study was 19.9 mg/kg, whereas the other two studies used lower doses, resulting in much lower C_max_ values of amikacin, which may not have been sufficient to eliminate the infection. On the other hand, the higher doses reported in our study result in high minimum serum concentrations (mean C_min_ value of ~15 mg/L in cohorts #1 and #2, Table 2), indicating potential nephrotoxicity. Considering the target amikacin PK/PD for efficacy and safety, it is urgent to develop tools for amikacin dosage regimen stratification or individualization in critically ill cancer patients with renal dysfunction. In this sense, we proposed a mechanistic model for study and proposed more rational amikacin dose regimens in this population.

There is no guideline for amikacin dosage recommendation in oncologic patients with renal dysfunction. Observing the published guidelines for the general population, there are large discrepancies among them, as reviewed by Pérez-Blanco et al. [9]. Stratifying the general population based on CLcr value and albumin level, Pérez-Blanco et al. [9] demonstrated that a dosage regimen of 27.5 mg/kg (q24h) has a high percentage of efficacy target attainment (C_max_/MIC > 10, MIC of 4 mg/L) for all stratified groups evaluated. Nevertheless, the fraction for safety target attainment (C_min_ < 4 mg/L) is decreased in the groups with a higher degree of renal dysfunction. On the other hand, the lower doses (4–15 mg/kg) and higher frequency administrations (q36–48h) suggested by some guidelines, indicate a low efficacy target attainment [9]. In the present study, we applied the oncologic PBPK model to the MIPD strategy, simulating amikacin dosage regimens in critically ill cancer patients with renal dysfunction (Figure 4). As it is observed, the treatment success rate for some dosage regimens is low. This can be attributed to the high interindividual variability in amikacin exposure within the cancer population. For cancer patients with severe renal dysfunction (Figure 4), the lower dose simulated of 7.5 mg/kg (q24h) demonstrated a high efficacy target (AUC/MIC > 80, MIC of 4 mg/L) attainment > 80%, but low safety target attainment (C_min_ < 5 mg/L) rates. This is extremely concerning since high amikacin serum levels at the end of drug administration interval are related to high nephrotoxicity incidence [35].

In the goodness-of-fit plot (Figure 5) evaluating the model’s performance in dose individualization through the digital twin strategy, the data points are close to the diagonal line representing unity. Considering the decision criterion for acceptable PBPK model simulation as 2-fold or the stringent criterion of 1.25-fold of the observed measurement, 83.8% and 45% of the data attended the criterion, respectively, indicating a promising PBPK model for amikacin dose individualization.

The main limitation of our PBPK model, developed for critically ill cancer patients with varying degrees of renal dysfunction, is the absence of other pathophysiological factors related to cancer and inflammation in the model. In addition, 11–13% of the patients were under dialysis and their glomerular filtration rate was not properly studied; a different model including dialysis impact on amikacin clearance is necessary to elucidate this. Furthermore, the co-administered drugs and comorbidities were not included in the model. Including these covariates in the model could enhance the model’s performance and reduce unexplained variability.

The amikacin PBPK model developed will function as a dynamic tool. In the future, additional parameters associated with interindividual variability, such as co-administered nephrotoxic drugs and the patient’s level of inflammation, will be incorporated to enhance the model’s performance. This will ensure its continued use for optimizing dosing in hospitals. Although the current PBPK model shows potential for use in a MIPD strategy, the proposed amikacin dosage regimens should be applied with caution and continue to be guided by TDM.

## 5. Conclusions

The present study shows that critically ill cancer patients evaluated during TDM routine presented reduced amikacin clearance, independently of their renal function status. The robust amikacin TDM data were employed for the development, refinement, and validation of the PBPK model. To fit the reduced amikacin clearance in this population, the PBPK model integrated reduced eGFR values stratifying the cancer patients into mild or severe renal impairment groups. The validated PBPK model was employed as a MIPD tool for amikacin dose stratification and individualization in critically ill cancer patients.

Our approach has the potential to be utilized in amikacin dosage regimen stratification and individualization to achieve the efficacy PK/PD index and help to avoid toxicity during the hospitalization of critically ill cancer patients with renal dysfunction. Our MIPD approach demonstrates potential in optimizing amikacin dosing for critically ill cancer patients. However, it does not eliminate the need for TDM due to unexplained variability still not accounted for by the PBPK model.

## Figures and Tables

**Table 1:** Characteristics of digital cancer patients with different renal dysfunction degrees created for the final amikacin PBPK model applied for critically ill cancer patients’ precision dosing.

Physiological Parameters	Digital Cancer Patients		Source
	Cancer Patients with Mild Renal Impairment	Cancer Patients with Severe Renal Impairment	
Plasma protein ratio to HV	0.99	0.85	Heimbach [23]; Wu [22]
Hematocrit	0.47	0.40	Heimbach [23]; Wu [22]
eGFR (mL/min/1.73 m^2^)	63.12 *	18.35 *	Parameter Identification
Hepatic blood flow ratio to HV	1.00	0.37	Heimbach [23]; Wu [22]
Kidney volume (L)	0.37	0.16	Heimbach [23]; Wu [22]
Albumin ratio to HV	1.00	0.85	Heimbach [23]; Wu [22]

Abbreviations—HV: healthy volunteers. PI: parameter identification tool in PK-SIM for model refinement. eGFR: estimated glomerular filtration rate. * Coefficient variation for this parameter was set at 70% in the model.

**Table 2:** Clinical and demographic characteristics of critically ill cancer patients from internal and external validation expressed as percentage or mean (range).

Parameter	Data Internal Validation Cohort #1 (*n* = 46)	Data External Validation Cohort #2 (*n* = 98)
Female ratio (%)	37	40
Age (years)	65 (20–89)	65.5 (20–89)
Body Weight (kg)	75.8 (44.8–100.0)	74.3 (41.5–132)
Serum creatinine	1.36 (0.19–3.56)	1.21 (0.19–4.82)
Creatinine Clearance * (mL/min)	50.6 (13.9–302)	57.72 (14.00–304.5)
CRP mg/dL (24–48 h)	N.C.	16.13 (0.50–49.74)
Amikacin Dose (mg)	1400 (300–3000)	1225 (300–3000)
C_min_ (mg/L)	14.6 (0.8–39.4)	15.5 (0.8–65.4)
C_max_ (mg/L)	53.5 (12–125)	51.9 (9.9–172.5)
SOFA score	5 (0–17)	5 (0–17)
Arterial Hypertension (%)	47.8	39.0
Diabetes Melittus (%)	26.0	21.0
COPD (%)	10.9	5.0
Hearth failure (%)	2.2	4.0
Mechanical ventilation (%)	57.4	41.0
Dialysis (%)	13	11.0
Meropenem (%)	100	79.0
Vancomycin (%)	76.1	65.0
Polymyxin (%)	21.7	20.0
Furosemide (%)	87.0	77.0
Noradrenaline (%)	45.7	43.0

* Obtained by the Cockcroft–Gault formula. Abbreviations: SOFA score, sequential organ failure assessment score; COPD, chronic obstructive pulmonary disease; CRP, C-reactive protein; C_min_, serum concentration obtained 8 to 24 h after infusion end; C_max_: serum concentration obtained 30 min to 2 h after infusion end; N.C., Not collected.

**Table 3:** Individual characteristics of critically ill cancer patients (*n* = 40, Cohort #3) used in digital twin strategy employing amikacin PBPK model.

Patient ID	Gender	Age (Years Old)	Body Weight (kg)	Height (cm)	CLcr (mL/min)	Meropenem	Vancomycin	Polymixin	Furosemide	In-Hospital Mortality	Hypertension	Diabetes	Congestive Heart Failure	Surgery Last 15 Days	Noradrenaline	Mechanical Ventilation
	Cancer patients with Severe renal dysfunction															
1	M	67	110	175	21.0	1	1	0	1	Y	1	1	0	0	0	0
2	M	75	87.5	182	21.7	1	1	0	1	Y	0	0	0	0	0	0
3	W	20	43.6	153	22.3	1	1	0	1	Y	0	1	0	1	0	0
4	M	78	67.55	166	22.5	0	0	0	0	N	0	0	0	1	0	0
5	M	25	65.65	172	23.2	1	1	0	1	N	0	0	0	1	0	0
6	M	76	68.1	173	31.5	1	0	0	1	Y	1	0	0	1	0	0
7	W	79	57	162	33.6	0	0	0	1	Y	1	0	0	1	0	0
8	M	71	66.2	162	39.9	1	1	0	1	Y	1	0	0	0	1	1
9	W	69	81	168	39.9	1	1	0	0	Y	0	0	0	0	0	0
10	W	74	103	160	42.9	0	1	0	1	Y	0	0	0	1	1	0
11	M	25	72.5	172	43.7	1	1	0	1	Y	0	0	0	1	1	1
12	W	66	77.6	154	45.5	1	1	1	1	Y	1	0	0	1	0	0
13	W	84	57.5	167	52.1	1	1	0	1	Y	1	0	0	0	0	0
14	M	66	98	169	54.4	1	0	0	1	Y	1	0	0	0	1	0
15	W	57	96.8	158	54.8	1	1	0	1	N	1	1	0	1	0	0
16	W	69	48.3	160	56.2	1	1	0	1	N	0	0	0	1	1	0
17	W	68	97.5	165	56.4	1	1	0	1	Y	0	0	0	0	1	0
18	W	54	58.5	163	57.1	0	0	0	1	Y	0	0	0	0	0	1
19	M	68	63.55	170	58.3	1	1	0	1	N	1	1	0	0	0	1
20	W	44	92	167	58.9	1	1	1	1	Y	0	0	0	1	0	1
	Cancer patients with Mild renal dysfunction															
21	W	68	43.95	169	63.3	0	0	0	0	N	0	0	0	0	0	0
22	W	68	45.65	169	68.1	0	0	0	1	N	0	0	0	0	0	0
23	W	73	78.1	156	71.8	0	1	0	1	Y	1	1	0	1	1	1
24	M	60	65.6	150	80.1	1	1	0	0	N	0	0	0	1	0	0
25	M	80	68.7	178	83.0	1	0	0	0	Y	0	0	0	1	1	1
26	M	77	51	153	85.8	0	0	0	1	N	0	0	0	0	0	0
27	W	81	69	149	90.7	1	0	0	1	Y	0	0	0	1	0	0
28	W	55	71.9	159	91.3	1	1	0	0	N	0	0	0	0	0	0
29	W	53	50.1	150	105.0	0	0	0	0	N	0	0	0	1	0	0
30	M	54	100.5	185	105.3	1	0	0	1	Y	1	0	0	1	0	0
31	M	64	78	169	111.3	1	1	0	0	Y	0	0	0	1	1	1
32	M	65	51.5	178	111.8	1	0	0	1	N	0	0	0	0	1	0
33	M	56	83	178	112.6	1	1	0	1	N	0	0	0	0	0	0
34	W	51	53.5	158	127.8	1	0	0	1	Y	0	0	0	1	0	0
35	M	29	106.5	182	129.3	0	0	0	0	N	1	0	0	1	0	0
36	M	59	114	180	135.0	1	1	0	1	N	1	0	0	1	0	0
37	M	26	56.5	179	149.1	1	1	1	0	Y	0	0	0	0	0	0
38	W	55	61.4	160	157.6	1	1	0	1	N	0	0	0	0	0	1
39	M	51	66.5	173	228.3	0	0	0	0	N	0	0	0	1	0	0
40	M	72	115	122	236.1	1	0	0	1	Y	0	0	0	0	0	0

Abbreviations: M—Man; W—woman; CLcr—Creatinine clearance (calculated with Cockroft–Gault formula); Y—Yes; N—No. The binary categorical value 0 corresponds to no for coadministration drug or comorbidity, and 1 corresponds to yes for coadministration drug or comorbidity.

## Data Availability

The data sets obtained and/or analyzed during the current study are available from the corresponding author on reasonable request.

## Supplementary Materials

The following supporting information can be downloaded at: https://www.mdpi.com/article/10.3390/pharmaceutics17030297/s1: Table S1. Summary of input data for amikacin physiologically based pharmacokinetic model using PK-SIM version 11.3.; Table S2. Clinical studies in healthy and renal patients used for validation of the base model in healthy volunteers and patients with different renal dysfunction degree.; Table S3. Characteristics of virtual individuals created in PK-Sim for the amikacin base model using renal function classification according to FDA (2024) [17]; Figure S1. Estimated Glomerular Filtration Rate (eGFR) distribution in two virtual cancer population groups according to renal dysfunction in the final amikacin PBPK model; Figure S2. Amikacin plasma concentration versus time profiles validating the amikacin PBPK base model in healthy volunteers; Table S4. Comparison of observed and predicted amikacin pharmacokinetic parameters in healthy volunteers; Figure S3. Amikacin plasma concentration versus time profiles validating the amikacin PBPK base model in non-oncologic patients with renal dysfunction; Table S5. Comparison of observed and predicted amikacin pharmacokinetic parameters in non-oncologic patients with renal dysfunction; Figure S4. Results of sensitivity analysis for amikacin systemic clearance for input parameters considering the PBPK model in developing for critically ill cancer patients with different severities of renal dysfunction; Figure S5. Panels of individual concentration versus time profile of virtual twins predicted (solid line) with amikacin final PBPK model and observed data (blue closed circles) from Therapeutic Drug Monitoring.

## References

[1] Ramirez M.S., Tolmasky M.E. (2017). Amikacin: Uses, Resistance, and Prospects for Inhibition. Molecules.

[2] Pyle-Eilola A.L., Dasgupta A. (2024). Chapter 8—Guidelines for monitoring vancomycin, aminoglycosides, and other antibiotics. Therapeutic Drug Monitoring.

[3] Chan K., Ledesma K.R., Wang W., Tam V.H. (2020). Characterization of Amikacin Drug Exposure and Nephrotoxicity in an Animal Model. Antimicrob. Agents Chemother..

[4] Abdul-Aziz M.H., Alffenaar J.C., Bassetti M., Bracht H., Dimopoulos G., Marriott D., Neely M.N., Paiva J.A., Pea F., Sjovall F. (2020). Antimicrobial therapeutic drug monitoring in critically ill adult patients: A Position Paper. Intensive Care Med..

[5] Agence française de sécurité sanitaire des produits de santé (2012). Update on good use of injectable aminoglycosides, gentamycin, tobramycin, netilmycin, amikacin. Pharmacological properties, indications, dosage, and mode of administration, treatment monitoring. Med. Mal. Infect..

[6] Garraffo R., Drugeon H.B., Dellamonica P., Bernard E., Lapalus P. (1990). Determination of optimal dosage regimen for amikacin in healthy volunteers by study of pharmacokinetics and bactericidal activity. Antimicrob. Agents Chemother..

[7] Marsot A., Guilhaumou R., Riff C., Blin O. (2017). Amikacin in critically Ill patients: A review of population pharmacokinetic studies. Clin. Pharmacokinet..

[8] Telles J.P., Diegues M.S., Migotto K.C., de Souza Borges O., Reghini R., Gavazza B.V., Pinto L., Caruso P., e Silva I.L.F., Schmidt S. (2023). Failure to predict amikacin elimination in critically ill patients with cancer based on the estimated glomerular filtration rate: Applying PBPK approach in a therapeutic drug monitoring study. Eur. J. Clin. Pharmacol..

[9] Pérez-Blanco J.S., Sáez-Fernández E.M., Calvo M.V., Lanao J.M., Martín-Suárez A. (2021). Evaluation of Current Amikacin Dosing Recommendations and Development of an Interactive Nomogram: The Role of Albumin. Pharmaceutics.

[10] (2024). European Committee on Antimicrobial Susceptibility Testing. Clinical Breakpoints and Dosing of Antibiotics. https://www.eucast.org/fileadmin/src/media/PDFs/EUCAST_files/Breakpoint_tables/v_14.0_Breakpoint_Tables.pdf.

[11] Gilbert D.N., Chambers D.N., Eliopoulos G.M., Saag M.S., Pavia A.T., Black D., Freedman D.O., Kim K., Schwartz B.S. (2019). The Sanford Guide to Antimicrobial Therapy 2019: 50 Years: 1969–2019.

[12] Medication Safety Queensland. Aminoglycoside Dosing in Adults. Department of Health. https://www.health.qld.gov.au/__data/assets/pdf_file/0019/713323/aminoglycoside-guidelines.pdf.

[13] Mensa J., Soriano A., García-Sánchez J.E., Letang E., López-Suñé E., Marco F., Llinares E., Barberán J. (2020). Therapeutic Guide Mensa 2020.

[14] Taylor Z.L., Poweleit E.A., Paice K., Somers K.M., Pavia K., Vinks A.A., Punt N., Mizuno T., Girdwood S.T. (2023). Tutorial on model selection and validation of model input into precision dosing software for model-informed precision dosing. CPT Pharmacomet. Syst. Pharmacol..

[15] Polasek T.M., Rostami-Hodjegan A. (2020). Virtual Twins: Understanding the Data Required for Model-Informed Precision Dosing. Clin. Pharmacol. Ther..

[16] Mostafa S., Polasek T.M., Bousman C., Rostami-Hodjegan A., Sheffield L.J., Everall I., Pantelis C., Kirkpatrick C.M.J. (2023). Delineating gene-environment effects using virtual twins of patients treated with clozapine. CPT Pharmacomet. Syst. Pharmacol..

[17] Food and Drug Administration. Pharmacokinetics in Patients with Impaired Renal Function—Study Design, Data Analysis, and Impact on Dosing Guidance for Industry. https://www.fda.gov/regulatory-information/search-fda-guidance-documents/pharmacokinetics-patients-impaired-renal-function-study-design-data-analysis-and-impact-dosing.

[18] Lode H., Grunert K., Koeppe P., Langmaack H. (1976). Pharmacokinetic and clinical studies with amikacin, a new aminoglycoside antibiotic. J. Infect Dis..

[19] Delattre I.K., Musuamba F.T., Nyberg J., Taccone F.S., Laterre P.F., Verbeeck R.K., Jacobs F., Wallemacq P.E. (2010). Population pharmacokinetic modeling and optimal sampling strategy for Bayesian estimation of amikacin exposure in critically ill septic patients. Ther. Drug Monit..

[20] Mahmoudi L., Mohammadpour A.H., Ahmadi A., Niknam R., Mojtahedzadeh M. (2013). Influence of sepsis on higher daily dose of amikacin pharmacokinetics in critically ill patients. Eur. Rev. Med. Pharmacol. Sci..

[21] De Winter S., van Hest R., Dreesen E., Annaert P., Wauters J., Meersseman W., Van den Eede N., Desmet S., Verelst S., Vanbrabant P. (2021). Quantification and Explanation of the Variability of First-Dose Amikacin Concentrations in Critically Ill Patients Admitted to the Emergency Department: A Population Pharmacokinetic Analysis. Eur. J. Drug Metab. Pharmacokinet..

[22] Wu C., Liu H., Yu S., Ren C., Zhang J., Wang G., Li B., Liu Y. (2022). Prediction of pharmacokinetics and pharmacodynamics of trelagliptin and omarigliptin in healthy humans and in patients with renal impairment using physiologically based pharmacokinetic combined DPP-4 occupancy modeling. Biomed. Pharmacother..

[23] Heimbach T., Chen Y., Chen J., Dixit V., Parrott N., Peters S.A., Poggesi I., Sharma P., Snoeys J., Shebley M. (2021). Physiologically-Based Pharmacokinetic Modeling in Renal and Hepatic Impairment Populations: A Pharmaceutical Industry Perspective. Clin. Pharmacol. Ther..

[24] Saravi B., Goebel U., Hassenzahl L.O., Jung C., David S., Feldheiser A., Stopfkuchen-Evans M., Wollborn J. (2023). Capillary leak and endothelial permeability in critically ill patients: A current overview. Intensive Care Med. Exp..

[25] Ferreira A., Martins H., Oliveira J.C., Lapa R., Vale N. (2021). PBPK Modeling and simulation of antibiotics amikacin, gentamicin, tobramycin, and vancomycin used in hospital practice. Life.

[26] Wu M., Feng K., Wu X., Liu C., Zhu S., Martins F.S., Yu M., Lv Z., Yan M., Sy S.K.B. (2024). Prediction of tissue exposures of polymyxin-B, amikacin and sulbactam using physiologically-based pharmacokinetic modeling. Front. Microbiol..

[27] Meraz-Munoz A., Langote A.D., Jhaveri K., Izzedine H., Gudsoorkar P. (2021). Acute Kidney Injury in the Patient with Cancer. Diagnostics.

[28] Soares M., Salluh J.I., Carvalho M.S., Darmon M., Rocco J.R., Spector N. (2006). Prognosis of critically ill patients with cancer and acute renal dysfunction. J. Clin. Oncol..

[29] Liu C., Wei W., Yang L., Li J., Yi C., Pu Y., Yin T., Na F., Zhang L., Fu P. (2023). Incidence and risk factors of acute kidney injury in cancer patients treated with immune checkpoint inhibitors: A systematic review and meta-analysis. Front. Immunol..

[30] Libório A.B., Abreu K.L., Silva G.B., Lima R.S., Barreto A.G., Barbosa O.A., Daher E.F. (2011). Predicting hospital mortality in critically ill cancer patients according to acute kidney injury severity. Oncology.

[31] Wei M., Huang M., Duan Y., Wang D., Xing X., Quan R., Zhang G., Liu K., Zhu B., Ye Y. (2023). Prognostic and risk factor analysis of cancer patients after unplanned ICU admission: A real-world multicenter study. Sci. Rep..

[32] Lameire N.H., Flombaum C.D., Moreau D., Ronco C. (2005). Acute renal failure in cancer patients. Ann. Med..

[33] Nakayama H., Suzuki M., Usuki K., Kato T. (2019). Amikacin Pharmacokinetics in Terminal Stage of Hematological Malignancy. Ther. Drug Monit..

[34] Aquino M., Tinoco M., Bicker J., Falcão A., Rocha M., Fortuna A. (2023). Therapeutic Drug Monitoring of Amikacin in Neutropenic Oncology Patients. Antibiotics.

[35] Bertino J.S., Booker L.A., Franck P.A., Jenkins P.L., Franck K.R., Nafziger A.N. (1993). Incidence of and significant risk factors for aminoglycoside-associated nephrotoxicity in patients dosed by using individualized pharmacokinetic monitoring. J. Infect. Dis..

