# Supplementary material for: Amikacin Dosing Adjustment in Critically Ill Oncologic Patients: A Study with Real-World Patients, PBPK Analysis, and Digital Twins
Source: Pharmaceutics. 2025 Feb 24;17(3):297. doi: 10.3390/pharmaceutics17030297 (PMC11944554; doi:10.3390/pharmaceutics17030297)
Supplement: Supplementary file 1 [file pharmaceutics-17-00297-s001.zip › pharmaceutics-3468705-supplementary.pdf]

Supplementary material

Amikacin dosing adjustment in critically ill oncologic patients: a study with real-world patients, PBPK analysis and digital twins

Table S1: Summary of input data for amikacin physiologically based pharmacokinetic model using PK-SIM version 11.3.

**Parameter**	**Value**	**Method/Reference**
**Physicochemical properties**		
Molecular weight (g/mol)	585.61	Drugbank
log *P*	-8.6	Drugbank
pKa	8.1	Drug Bank
fraction unbound (fu)	0.9	DrugBank
Solubility at pH 7 (mg/mL)	50	Drugbank
**Distribution**		
Model		
Partition Coefficients	Rodgers and Rowland	Calculated with PK-Sim
Cellular permeabilities	PK-Sim standard	Calculated with PK-Sim
Specific organ permeability (cm/min)	1.79E-15	Calculated with PK-Sim for healthy volunteers and non-cancer patients
Specific organ permeability (cm/min)	1.79E-06	Fitted with Parameter Identification tool for cancer patients
**Elimination**		
Glomerular Filtration Rate	1.0	

Table S2: Clinical studies in healthy and renal patients used for validation of the base model in healthy volunteers and patients with different renal dysfunction degree.

Reference	n	women	age (years)	weight (kg)	Infusion time (min)	single dose	CL (L/h)	CLrenal (L/h)	Creatinine clearance (mL/min)	GFR classification[a]	Disease
								**Healthy volunteers**			
Garrafo et al. [6]	6	0.5	23-31	50-75	30	7.5 mg/kg	7.6	-		Normal	
Garrafo et al. [6]	6	0.5	23-31	50-75	30	15 mg/kg	6.77	-		Normal	
Lode et al. [18]	12	0.5	30	65.5	60	7.5 mg/kg	6.5	6.1		Normal	
								**Renal Patients**			
Delattre et al. [19]	88	0.35	22-89	38-125	30	25 mg/kg	2.21	-	55.5 (12.3 – 408)	Moderate	ICU sepsis
Mahmoudi et al. [20]	30	0.3	44.43 (±14)	69.47 (±9.7)	60	1500 mg	3.88	-	69 (±7.53)	Mild	ICU sepsis
De winter et al. [21]	48	0.36	73 (62-81)	70 (60 – 85)	30	15 mg/kg	1.8	-	38.4 (26.5 –62.9)	Moderate	Critically Ill Patients Admitted to the Emergency Department
De winter et al. [21]	49	0.36			30	20 mg/kg	1.8	-	38.4 (26.5 –62.9)	Mild	

a Glomerular Filtration rate (GFR) classification according to FDA (2024) [17] guidance.

Table S3: Characteristics of virtual individuals created in PK-Sim for the amikacin base model using renal function classification according to FDA (2024) [17].

Physiological parameters		Disease patients by renal dysfunction stage			Source
	**Healthy Volunteer (HV) ≥ 90 mL/min**[b]	**Mild 60 – 89 mL/min**	**Moderate 30- 59 mL/min**	**Severe ≤ 29 mL/min**	
Plasma protein ratio to HV	1.00	0.99	0.93	0.85	Heimbach et al. [23]; Wu et al. [22]
Hematocrit	0.47	0.47	0.44	0.40	Heimbach et al. [23]; Wu et al. [22]
Renal blood flow ratio to HV (eGFR mL/min/m^2)[a]	1.00 (117)	0.7 (70)	0.50 (40)	0.47 (20)	Heimbach et al. [23]; Wu et al. [22]
Hepatic blood flow ratio to HV	1.00	1.00	1.00	0.37	Heimbach et al. [23]; Wu et al. [22]
Kidney volume (L)	0.44	0.37	0.30	0.16	Heimbach et al. [23]; Wu et al. [22]
Albumin ratio to HV	1.00	1.00	0.93	0.85	Heimbach et al. [23]; Wu et al. [22]

[a] eGFR mean value for the corresponding virtual population; b Renal Function classification according to FDA (2024) [17] for dedicated renal impairment studies.

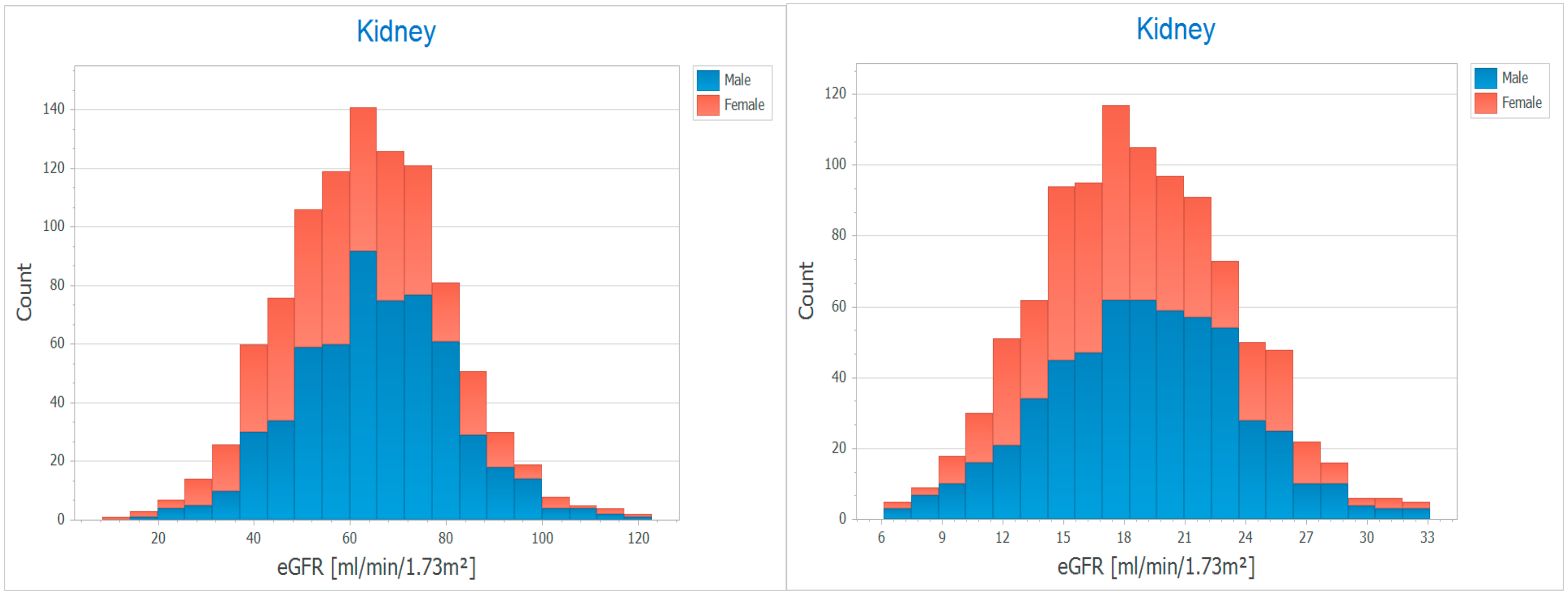

Figure S1: Estimated Glomerular Filtration Rate (eGFR) distribution in two virtual cancer population groups according to renal dysfunction in the final amikacin PBPK model. **The left panel** represents critically ill cancer patients with normal to mild renal dysfunction stages**. The right panel** represents critically ill cancer patients with moderate to severe renal dysfunction stages.

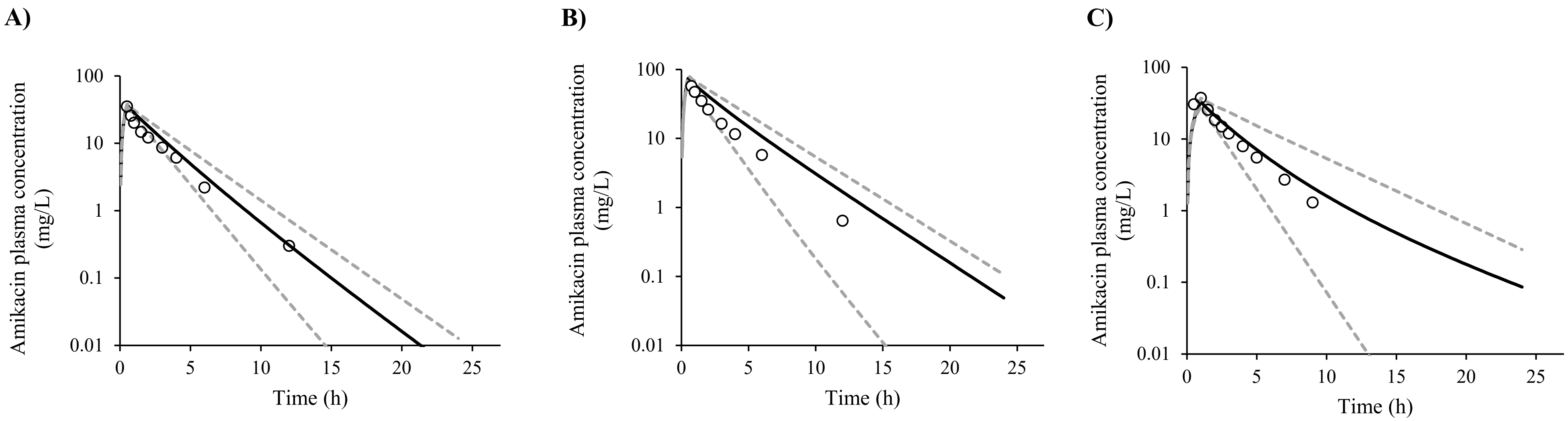

Figure S2: Amikacin plasma concentration versus time profiles validating the amikacin PBPK base model in healthy volunteers. Open circles represent observed in vivo data from the following studies: **A)** Garrafo et al. [6], 7.5 mg/kg**; B)** Garrafo et al. [6] 15 mg/kg; **C)** Lode et al. [18]; 7.5 mg/kg. The solid line represents the simulated mean plasma concentration and the dashed lines represent the simulated 5th and 95th percentiles with the base amikacin PBPK model.

Table S4: Comparison of observed and predicted amikacin pharmacokinetic parameters in healthy volunteers. The 0.5 to 2-fold observed/predicted ratio window is considered ideal for PBPK model quality evaluation.

	**Healthy volunteers**		
**Reference**	**Garrafo et al. [6]**	**Garrafo et al. [6]**	**Lode et al. [18]**
Parameter	7.5 mg/kg	15 mg/kg	7.5 mg/kg
CLobs (L/h)	7.6	6.8	4.3
CLpred (L/h)	5.6	5.0	5.2
Ratio CLobs/CLpred	1.3	1.4	0.8
Vdobs (L)	17.8	18.0	11.9
Vdpred (L)	11.9	11.9	12.4
Ratio Vdobs/Vdpred	1.5	1.5	0.9
C_{max}obs (mg/L)	35.1	76.0	37.5
C_{max}pred (mg/L)	36.3	74.7	32.6
Ratio C_{max}obs/C_{max}pred	0.9	1.0	1.1
AUCobs (mgh/L)	66.6	154.5	140.4
AUCpred (mgh/L)	90.2	225.6	107.1
Ratio AUCobs/AUCpred	0.7	0.7	1.3

Abbreviations: CL: clearance, Vd: Volume of distribution, AUC: Area Under the Curve, obs: observed data, pred: predicted data.

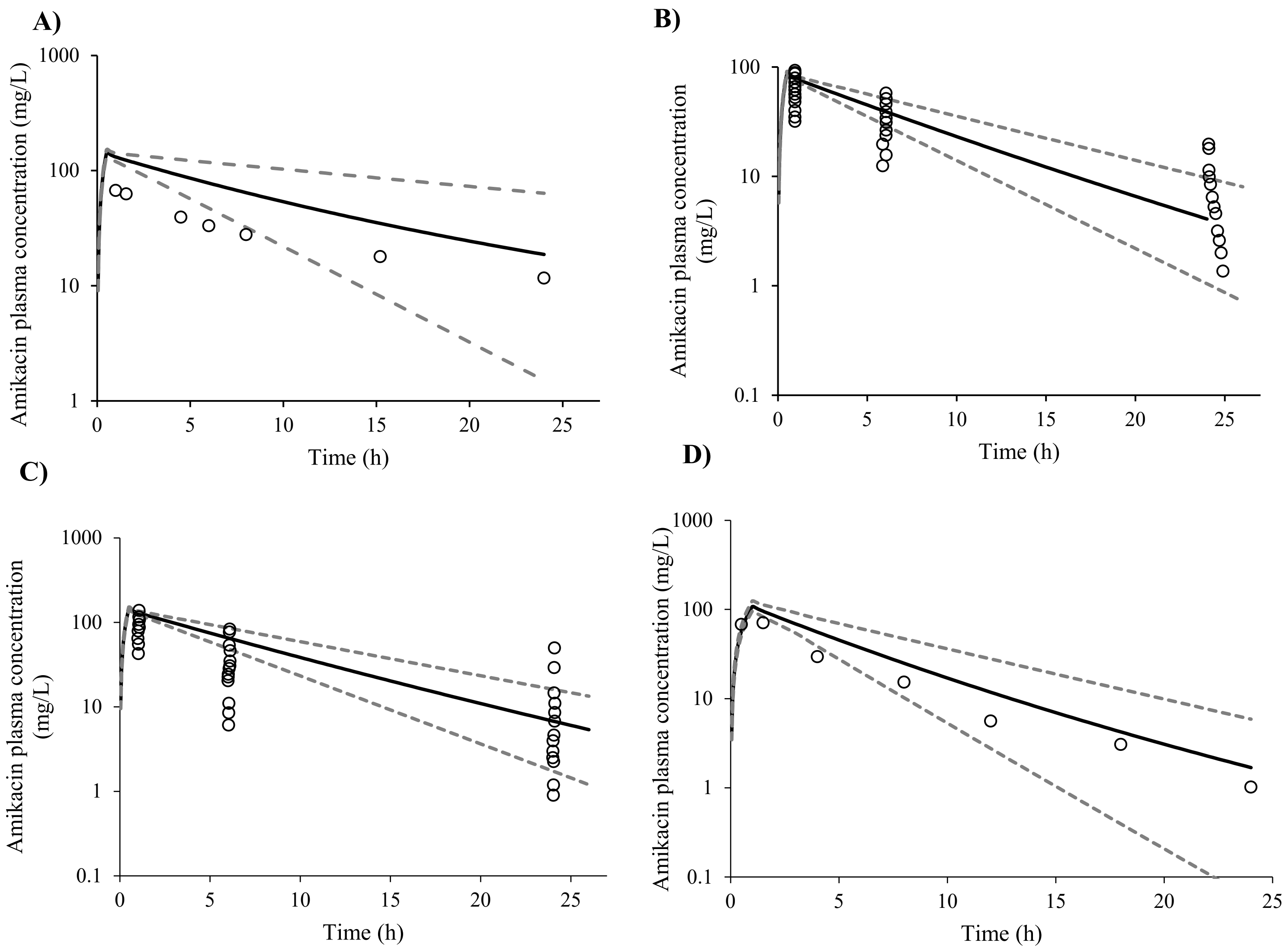
A)
Amikacin plasma concentration (mg/L)
1000
100
10
1
0
5
10
15
20
25
Time (h)
B)
Amikacin plasma concentration (mg/L)
100
10
1
0.1
0
5
10
15
20
25
Time (h)
C)
Amikacin plasma concentration (mg/L)
1000
100
10
1
0.1
0
5
10
15
20
25
Time (h)
D)
Amikacin plasma concentration (mg/L)
1000
100
10
1
0.1
0
5
10
15
20
25
Time (h)

Figure S3: Amikacin plasma concentration versus time profiles validating the amikacin PBPK base model in non-oncologic patients with renal dysfunction. Open circles represent observed in vivo data from the following studies: **A)** Dellatre et al. [19], 25mg/kg; **B)** De Winter et al. [21], 15 mg/kg; **C)** De Winter et al. [21], 25 mg/kg; **D)** Mahmoudi et al. [20], 1500 mg. The solid line represents the simulated mean plasma concentration and the dashed lines represent the simulated 5th and 95th percentiles with the base amikacin PBPK model.

Table S5: Comparison of observed and predicted amikacin pharmacokinetic parameters in non-oncologic patients with renal dysfunction. The 0.5 to 2-fold observed/predicted ratio window is considered ideal for PBPK model quality evaluation.

	**Patients with renal dysfunction**			
**Reference**	**Delattre et al. [19]**	**Mahmoudi [20]**	**De Winter [21]**	**De Winter [21]**
Parameters	25 mg/kg	1500 mg	15 mg/kg	25 mg/kg
CL_{obs} (L/h)	2.2	3.8	1.8	1.8
CL_{pred} (L/h)	1.6	1.4	1.4	1.4
Ratio CL_{obs}/CL_{pred}	1.4	2.8	1.3	1.3
Vd_{obs} (L)	28.5	25.0	29.0	29.0
Vd_{pred} (L)	14.7	12.6	11.9	11.9
Ratio Vd_{obs}/Vd_{pred}	1.9	1.9	2.4	2.4
$C_{max}obs$ (mg/L)	NC	71.5	NC	NC
$C_{max}pred$ (mg/L)	NC	108.0	NC	NC
Ratio $C_{max}obs/C_{max\ pred}$	NC	0.6	NC	NC
AUC_{obs} (mgh/L)	791.9	394.2	583.3	972.2
AUC_{pred} (mgh/L)	1785.6	586.5	653.8	1089.6
Ratio AUC_{obs}/AUC_{pred}	0.4	0.7	0.9	0.9

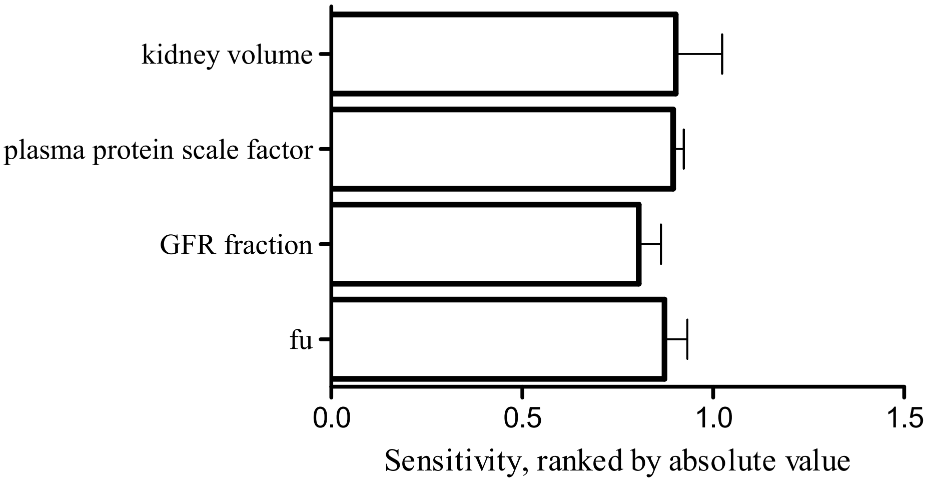

Figure S4: Results of sensitivity analysis for amikacin systemic clearance for input parameters considering the PBPK model in developing for critically ill cancer patients with different severities of renal dysfunction. Abbreviations: GFR: glomerular Filtration Rate; fu: plasma protein fraction unbound.

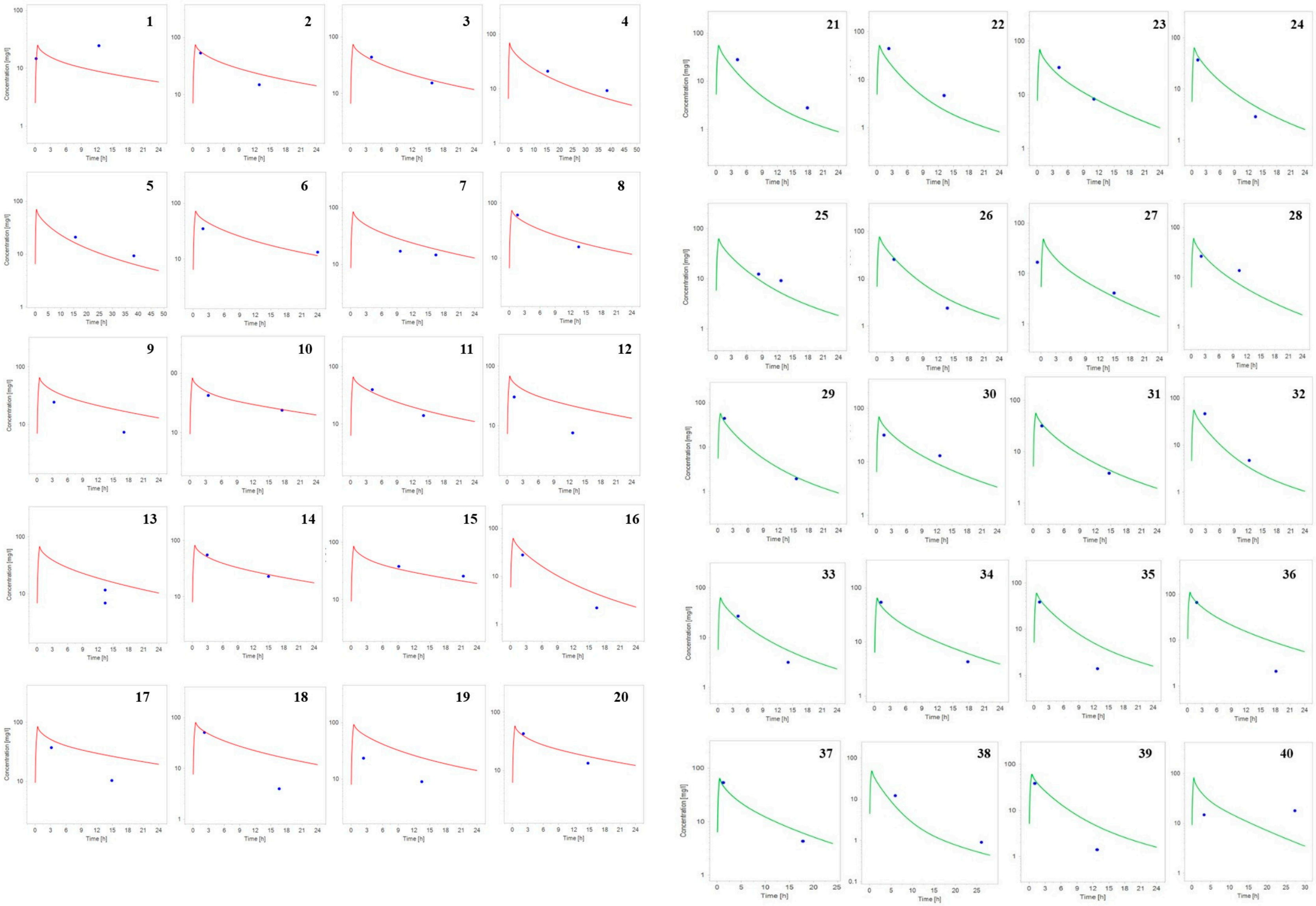
1
2
3
4
5
6
7
8
9
10
11
12
13
14
15
16
17
18
19
20
21
22
23
24
25
26
27
28
29
30
31
32
33
34
35
36
37
38
39
40
Concentration [mg/l]
Time [h]

Figure S5: Panels of individual concentration versus time profile of virtual twins predicted (solid line) with amikacin final PBPK model and observed data (blue closed circles) from Therapeutic Drug Monitoring. Green solid lines represent critically ill cancer patients with Mild Renal Impairment and Red solid lines represent critically ill Cancer patients with Severe Renal Impairment.

Footnote: For the PBPK simulations, the gender, age, body weight, and specific values for each virtual twin were considered. Renal function was classified for each patient depending on the creatinine clearance value calculated by Cockroft-Gault formula and considering the cutoff point of creatinine clearance (CLcr) value of 60 mL/min: Cancer patients with mild renal impairment model was used for critically ill cancer patients with a CLcr value of < 60 mL/min, while the Cancer patients with severe renal impairment model was used for critically ill cancer patients with a CLcr value ≥ 60 mL/min.
